# Variable processing and presentation of HIV epitopes in dendritic cells and macrophages to CD8 T cells

**DOI:** 10.1186/1742-4690-9-S2-P266

**Published:** 2012-09-13

**Authors:** P Gourdain, J Dinter, N Lai, M Shimada, E Duong, T Zhu, E Bracho-Sanchez, P Liebesny, S Le Gall

**Affiliations:** 1 The Ragon Institute of MGH, MIT and Harvard, Charlestown, MA, USA

## Background

Whether HIV-infectable subsets, such as CD4 T cells, monocytes, macrophages and dendritic cells (DCs), have equivalent capacity to produce and present MHC-I restricted epitopes to HIV-specific CD8 T cells is unknown. MHC-I epitopes are processed by an intracellular degradation pathway involving multiple proteases. In this study we analyzed the effect of toll-like receptor (TLR) agonist-mediated maturation on the processing and presentation of HIV antigens in monocyte-derived DCs and macrophages.

## Methods

Proteolytic activities were measured with a fluorescence-based activity assay. Cytosolic extracts were used as a source of peptidases to degrade extended epitopes in vitro. Degradation products were then analyzed by mass spectrometry and antigenicity was tested by a ^51^Cr release assay and a real-time killing assay.

## Results

Upon maturation with LPS or R848, the proteasomal and lysosomal activities in matured macrophages are significantly higher compared to matured DCs. The proteasomal tryptic and caspase-like activities as well as the lysosomal activities decreased approximately 1.5-fold in matured DCs. The degradation of the N-terminal extended epitope 3-ISW9 in LPS-matured DCs yielded 2-fold more optimal epitope ISW9 than in immature DCs, which resulted in a 3-fold higher cell lysis in a ^51^Cr release assay. In addition, the cross-presentation of exogenously added p24 protein by DCs or macrophages showed reduced killing of APCs by ISW9-specific CTLs compared to CTLs specific for TW10 or KF11. This lower ISW9-specific killing was partly rescued by preincubation with protease inhibitor.

## Conclusion

We showed that differences in antigen processing activities in DCs and macrophages upon maturation, and differences in HIV sequences sensitivity to intracellular degradation may affect the production and presentation of epitopes and thus the capacity of HIV-specific CTLs to recognize and kill infected cells. For the design of a vaccine immunogen it is critical to identify factors regulating the processing and presentation of epitopes.

Supported by NIH NIAID R01 A1084753.

